# Genomic insights into familial adenomatous polyposis: unraveling a rare case with whole *APC* gene deletion and intellectual disability

**DOI:** 10.1038/s41439-024-00270-3

**Published:** 2024-03-29

**Authors:** Hiroki Tanabe, Masami Ijiri, Kenji Takahashi, Honoka Sasagawa, Tomomi Kamanaka, Shohei Kuroda, Hiroki Sato, Takeo Sarashina, Yusuke Mizukami, Yoshio Makita, Toshikatsu Okumura

**Affiliations:** 1 Oncology Center, Asahikawa Medical University Hospital, Asahikawa, Japan; 2 Genetic Oncology Department, Asahikawa Medical University Hospital, Asahikawa, Japan; 3 Department of Genetic Counseling, Asahikawa Medical University Hospital, Asahikawa, Japan; 4 Department of Gastroenterology, Japanese Red Cross Asahikawa Hospital, Asahikawa, Japan; 5 Department of Internal Medicine, Asahikawa Medical University, Asahikawa, Japan

**Keywords:** Colon cancer, Genetic counselling

## Abstract

A young patient diagnosed with advanced colon cancer and liver metastasis was found to have familial adenomatous polyposis (FAP) through comprehensive genomic analysis. Whole-genome array comparative genomic hybridization (aCGH) revealed germline deletions at chromosome 5q22.1-22.2 encompassing the entire *APC* gene. The patient and her son exhibited mild intellectual disability without developmental delay. This case highlights the need for further exploration of the characteristics associated with whole *APC* deletions. aCGH is a valuable tool for studying FAP and provides a detailed analysis of large deletions.

## Case report

Familial adenomatous polyposis (FAP) is an autosomal dominant disorder resulting from germline mutations in the *APC* gene. The *APC* gene, comprising 15 exons and encoding a protein with 2843 amino acids, is implicated in ~80% of FAP cases^1^. Extensive genetic analysis has revealed germline variants in FAP patients, and most *APC* mutations are found in the 5’ half of the coding region. Genotype‒phenotype correlations have been reported for small-nucleotide alterations, including frameshift and nonsense mutations^2,3^. Large genomic deletions and duplications have been identified using multiplex ligation-dependent probe amplification (MLPA)^4^. Whole-genome array comparative genomic hybridization (aCGH) was used to identify a large deletion involving the middle portion of the long arm of chromosome 5^5^. Here, we report a case of an FAP patient with intellectual disability that was attributed to a large deletion involving 5q22.2.

The proband was a 28-year-old female who was referred to the emergency hospital with acute abdominal pain. Computed tomography (CT) demonstrated perforation of the descending colon, multiple colorectal polyps, multiple liver metastases and lymph node swelling. She underwent left hemicolectomy, and the subsequent histological diagnosis was moderately differentiated adenocarcinoma (pT4a, pStage IVa). Chemotherapy was selected for treatment of the residual metastasis. Colonoscopy revealed advanced colon cancer with multiple adenomatous polyps (>100). Head CT revealed an osteoma in her skull, and the phenotype was subsequently defined as Gardner’s syndrome.

The patient had slight intellectual disability without developmental delay or neurogenic abnormalities. She and her mother requested comprehensive genomic panel (CGP) analysis (OncoGuide^TM^ NCC oncopanel, Sysmex, Hyogo, Japan) of surgically resected colon cancer tissue after providing informed consent. This test can detect mutations in 124 genes and differentiate between germline and somatic mutations. The pathogenic mutations detected were *KRAS* G13D, *PIC3CA* H1047R, and *TP53* M169fs*2, but no targeted therapy was recommended by the expert panel. No germline findings were reported, but whole *APC* gene deletion was suspected due to the low amplicon depth of the *APC* gene in both the tumor tissue and blood samples (Fig. S1).

**Fig. 1:**
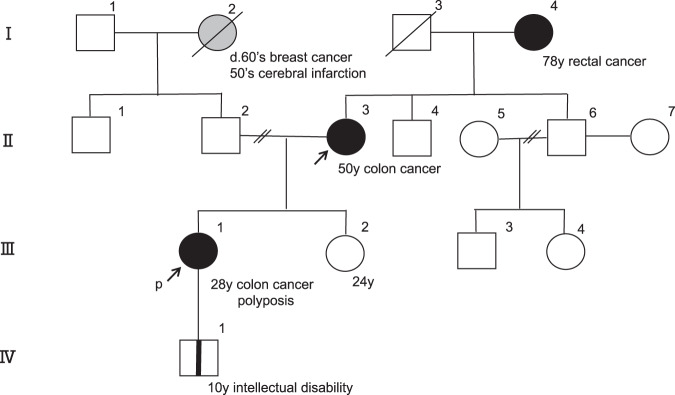
Pedigree of the patients. The arrow indicates the patients who underwent genetic counseling. A closed circle indicates an individual with colorectal cancer. Colorectal polyposis was observed in the proband (III-1) but not in her ancestors.

According to her familial history (Fig. 1), her mother (II-3) was treated for sporadic colon cancer. She refused genetic testing due to receiving cancer chemotherapy. Her son (IV-1), whose intelligence was slightly low, had a single-parent history because his father was not identified.

**Fig. 2:**
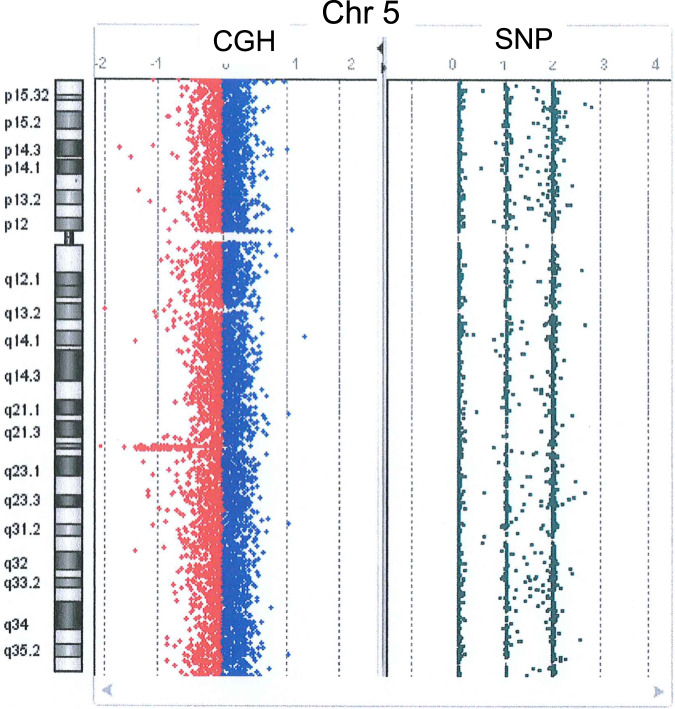
Comparative genomic hybridization analysis of chromosome 5. A heterozygous 5q22 deletion was detected. The minimal and maximal deletion positions in GRCh37 (start_stop) were 111143360_112213143 and 111118900_112239978, respectively.

After genetic counseling, aCGH (GenetiSure Dx Postnatal Assay, Agilent, Tokyo, Japan) was performed for further genetic testing. Notably, aCGH revealed the loss of chromosome 5 (chr5) q22.1-q22.2 (Fig. 2), the loss of chr3 p24.1-p23, and the gain of chr15 q15.3. The chr5 deletion included the entire *APC* gene (chr5:112043195-112181936 in GRCh37) located at 5q22.2 (Fig. S2), according to the Database of Chromosomal Imbalance and Phenotype in Humans Using Ensembl Resources (DECIPHER, https://www.deciphergenomics.org).

This case in which the entire *APC* gene was deleted, as determined by aCGH, is rare. Chromosome 5p22.1-22.2 deletion causes 1 Mb of heterozygous loss, including the *APC* gene, which was reported as a cytogenetically detected deletion in previous reports. Previously, karyotyping and fluorescence in situ hybridization were used to detect large submicroscopic genomic deletions, and aCGH was used to detect high-resolution copy number variants in whole chromosomes^6^. aCGH is sensitive and comprehensive, allowing detection of multiple variations, and annotations by specialists are needed. DECIPHER catalogs common copy number changes, enabling the identification of potentially pathogenic variants. aCGH can also be used for sequencing targeted genes. For FAP patients, germline *APC* variants are identified by direct sequencing using next-generation sequencing (NGS) and MLPA^5^. Sequencing has been used to detect *APC* gene variants, but ~20% of FAP patients do not carry these variants. MLPA is useful for detecting whole or large *APC* gene copy number variants in mutation-negative FAP patients. There are several case reports in which germline variants of FAP were examined via aCGH^7–10^.

Our young patient with advanced colon cancer derived from multiple colorectal polyposis was diagnosed with FAP according to the clinical features. A CGP was performed using NGS for cancer precision medicine in this patient. Because metastatic colon cancer is treated by chemotherapy, somatic genomic analysis with CGP was also conducted to determine the optimal chemotherapy regimen. Next, we used NGS to determine the sequence of 100 bp amplicons of 124 cancer-related genes from cancer tissue and peripheral blood. A large *APC* deletion was not detected by this targeted sequence, although both the somatic and germline amplicon depths of the *APC* gene were slightly low. A large number of *APC* variants have already been deposited in the ClinVar database (https://www.ncbi.nlm.nih.gov/clinvar/). For several FAP patients in which germline *APC* variants were not found, investigations of copy number variations have been performed. The genotype‒phenotype correlation of patients with chromosome 5q deletions has been discussed^10^. A classical FAP phenotype is associated with a mutation in codons 168–1250 or codons 1400–1580. A severe phenotype is caused by a mutation in codons 1250–1464. A more attenuated form is associated with mutations in three regions: the 5’ region of the *APC* gene, the alternative splicing region in exon 9, and the extreme 3’ end of the gene^11^.

Whole or partial *APC* gene deletions can be detected with recently developed genetic techniques^9,10,12^. MLPA and aCGH are candidates for confirming large deletions or duplications, and the latter genetic test was chosen for our patient. In our patient, two chromosomal losses and one gain were detected. The advantage of chromosomal analysis is that it can reveal unexpected genetic changes even in separate chromosomes. The CGH database includes some patients with large deletions in chromosomal region 5q22, including the *APC* gene. In a very recent case report, aCGH was utilized to identify a large 19.85 Mb deletion^12^. A case series with a literature review described a patient with intellectual disability and a colon neoplasm with an interstitial deletion of 5q identified by aCGH. Colorectal cancers are observed in some patients with 5q deletions, yet examination of colorectal polyposis in this context is limited. Among the primary dysmorphisms and symptoms linked to 5q deletions, the predominant manifestation identified in the analysis of 12 patients was mental retardation^12^. The cases documented in both the literature and the DECIPHER database are characterized by common clinical features, including predisposition to cancer, intellectual disability, and neurodevelopmental delay. Patients with these congenital changes should undergo genetic testing, including G-band, fluorescence in situ hybridization (FISH), and aCGH. aCGH offers high resolution, allowing for the detection of changes at the chromosomal level. This high sensitivity is particularly valuable when conventional methods, such as karyotyping or FISH, may not provide detailed information about genomic alterations. Moreover, this approach allows researchers and clinicians to explore potential genetic factors beyond the well-known *APC* genes. In the near future, long-read sequencing of large deletions may enable us to obtain detailed genomic information^13^. Additional clinical information is needed to establish the genotype‒phenotype correlations associated with the 5q22.2 deletion that includes the whole *APC* gene. The published cases have raised the question of whether whole *APC* deletion induces colorectal polyposis. Casper et al. reported a case of Gardner syndrome attributable to a substantial interstitial deletion of chromosome 5q, offering a comprehensive review of published cases^9^. Until 2014, 16 patients with FAP resulting from chromosome 5q deletions were documented, with all but one patient presenting with classic adenomatous polyposis rather than the profuse form. Most of these deletions were de novo alterations, consistent with our reported case in which the patient’s mother (II-3) exhibited sporadic colon cancer without polyposis. In the familial lineage (Fig. 1), our patient’s son (IV-1) carried a deletion in the 5q22.1-22.2 region, mirroring the genomic alteration of his mother (III-1). However, the genetic inheritance pattern of this large deletion is unclear. Meticulous follow-up of the young boy is important for addressing this issue.

In conclusion, this study describes a rare FAP patient characterized by a large deletion of chromosome 5q22.1-22.2 identified through comprehensive genomic analysis. The genetic variant was suspected by CGP and eventually identified by aCGH. These findings emphasize the importance of advanced genetic techniques in identifying complex genomic variations and suggest a need for additional research to elucidate the specific features associated with whole-*APC* gene deletions.

## Supplementary information

Supplementary figure

## Data Availability

The relevant data from this Data Report are hosted at the Human Genome Variation Database at 10.6084/m9.figshare.hgv.3376.
