# Supplementary material for: Genomic insights into familial adenomatous polyposis: unraveling a rare case with whole APC gene deletion and intellectual disability
Source: Hum Genome Var. 2024 Mar 29;11:13. doi: 10.1038/s41439-024-00270-3 (PMC10978947; doi:10.1038/s41439-024-00270-3)

**Figure S1**

Results of cancer genomic panel analysis. The depth of the *APC* gene was low in the tumor and blood samples.
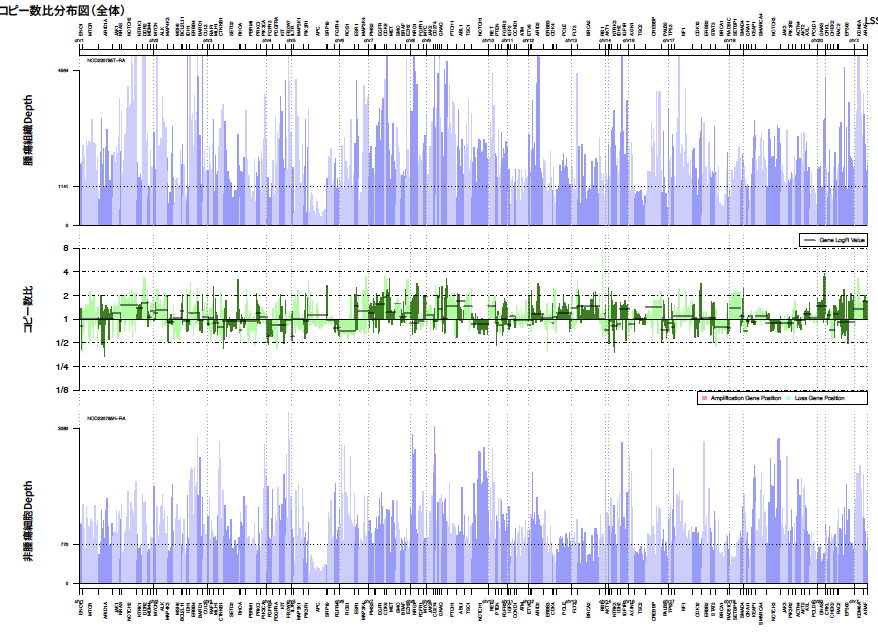


APC

APC

Depth (nontumor)

Copy number ratio

Depth (tumor)

**Figure S2**

The chromosome 5 deletion illustrated on DECIPHER website. The deletion of 5:111143360-112213143 is indicated by the gray area. The whole *APC* gene indicated by the red bar is included in this area.


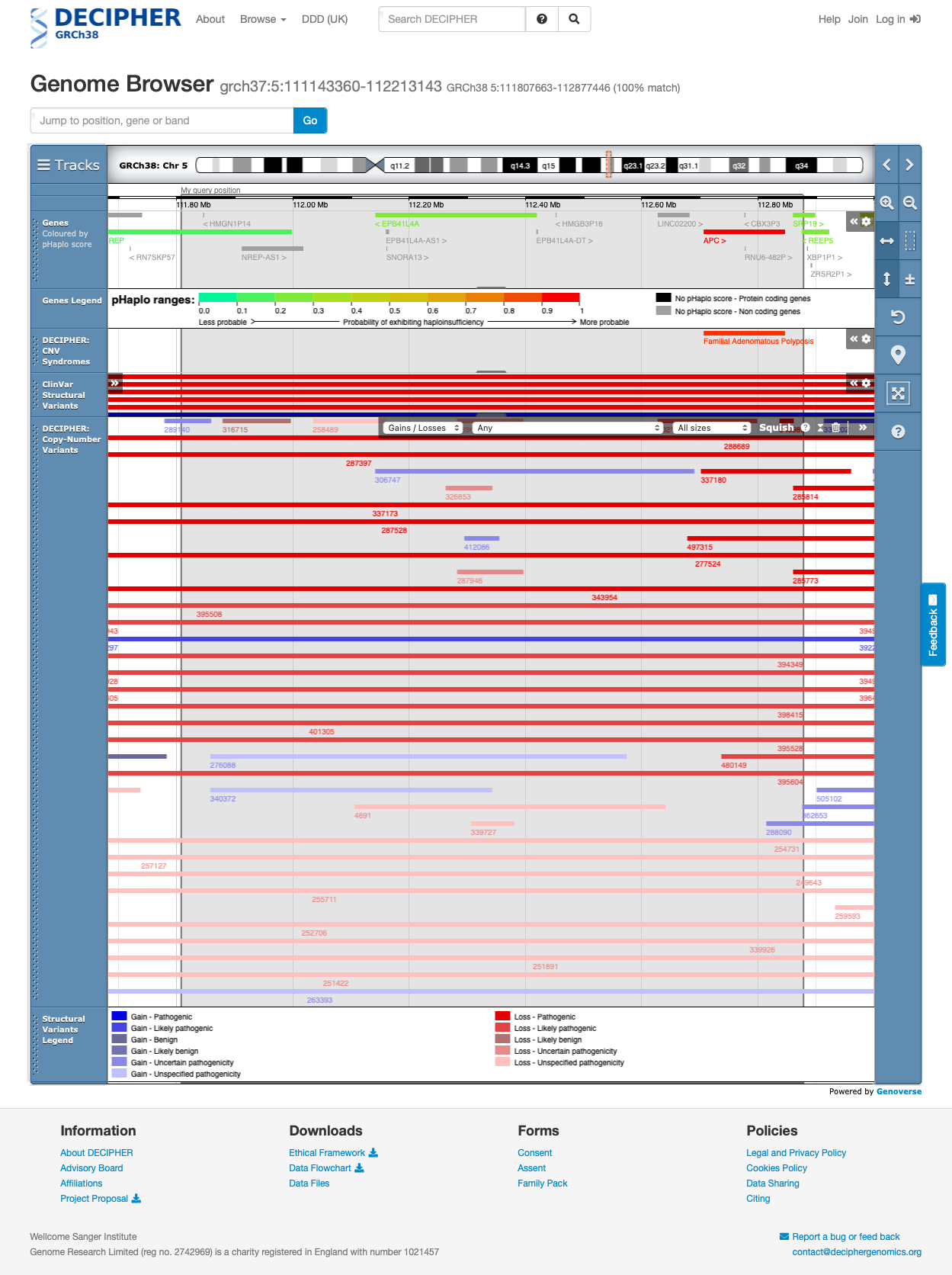

Supplement: Supplementary file 1 — Supplementary figure [file 41439_2024_270_MOESM1_ESM.docx]
